# Analyzing Nurse Sentiment in a Dutch Regional Newspaper During the COVID-19 Pandemic: Comparative Content Analysis of GPT-5, Gemini 2.5 Pro, and Human Coding

**DOI:** 10.2196/98374

**Published:** 2026-08-13

**Authors:** Bram van den Berkmortel, Daan Westra, Rachel Gifford, Frank Christian van de Baan

**Affiliations:** 1 Department of Health Services Research, Care and Public Health Research Institute, Maastricht University, Duboisdomein 30, Maastricht, Limburg, 6229GT, The Netherlands, 31 043 38 81 553

**Keywords:** AI, large language models, qualitative research, comparative study, health services research, COVID-19, natural language processing, sentiment analysis, medical informatics, nursing

## Abstract

**Background:**

Large language models (LLMs) are increasingly used in qualitative research, but their reliability compared to human analysis, especially on large, non-English datasets, is unclear. Previous studies on older models (like GPT-4) show limitations in nuance and token capacity.

**Objective:**

This study compared the qualitative analysis capabilities of OpenAI’s GPT-5 and Google’s Gemini 2.5 Pro (Gemini) with a human qualitative analysis. The study uses a large dataset of 317 Dutch newspaper articles from January 1, 2020, to December 31, 2023, investigating the sentiment toward nurses during the COVID-19 pandemic.

**Methods:**

The study used a 2-phase methodology. First, a thematic comparison was conducted where the human researchers, GPT-5, and Gemini independently generated inductive coding trees from the entire corpus. Second, a comparative test was performed where all 3 coders applied a predefined codebook to a 10% stratified random subsample. The human baseline was validated through double-coding by 2 independent researchers, achieving an acceptable intercoder reliability (α=0.703). The AI analysis was iterative, using model-optimized prompts and an article-by-article approach.

**Results:**

Both AI models successfully identified third-order themes (eg, “Health care heroes”) consistent with the data. In deductive application, however, both models systematically overcoded compared to the human consensus (181 and 183 codes vs 138), resulting in low intercoder reliability against the human baseline (α=0.487) for GPT-5 and (α=0.507) for Gemini.

**Conclusions:**

This study suggests a potential divergence in analytical logic. The observed coding frequencies indicate that LLMs may default to semantic presence (literal frequency), whereas human coders appear to prioritize interpretive significance (contextual weight), leading to systematic overcoding. Consequently, this article argues that LLMs should not be viewed as autonomous researchers but as high-sensitivity filtering instruments requiring human calibration. This study concludes that AI can serve as a valuable assistant for qualitative researchers. Still, it benefits from a rigorous, iterative, and human-in-the-loop approach to manage methodological friction and ensure nuanced, valid analysis.

## Introduction

There has been an increase in the use of AI in qualitative health care research [1]. With the rapid emergence of new large language models (LLMs) such as OpenAI’s GPT-5, AI is constantly improving [2]. These advancements have enabled machines to analyze vast amounts of text data and generate human-like responses, greatly enhancing the capabilities of AI in processing natural language [3]. It is therefore important to determine whether LLMs produce reliable results for interpretive social science, where reliability refers to consistency and transparency of the analytical process and to whether different analysts, human or AI, reach comparable interpretations when working from the same coding framework.

Recent literature confirms the potential of LLMs for qualitative analysis. However, it has also identified several profound limitations, which can be categorized into 3 distinct risks. First, there are significant concerns regarding accuracy and hallucination. A study by Li et al [4], which compared GPT-4 with human researchers in analyzing patient interviews, demonstrated only moderate agreement (Cohen κ=0.401). More critically, Wachinger et al [5] identified severe risks where AI models fabricated quotes not present in the original transcripts or “hallucinated” themes to fit a requested pattern, a phenomenon known as sycophancy. Second, models lack interpretive depth and reflexivity. While Li et al [4] found that AI could successfully identify major descriptive themes, human analysis provided a substantially richer diversity of subthemes. This suggests a gap between “diagnostic reliability” (the accurate retrieval of explicit factual statements) and “thematic reliability” (the consistent interpretation of latent meaning), where AI produces flat, sanitized descriptions that miss the profound human-driven nuances of health care narratives. Third, technical constraints have historically hindered holistic analysis. Both Li et al [4] and Wachinger et al [5] were constrained by token limits, forcing them to fragment data in ways that severed narrative arcs. While LLMs like GPT-4o show a balance of speed and accuracy in data processing, the consensus remains that AI functions best as a complementary tool rather than a replacement, as human expertise is essential for validating complex, ambiguous decisions [1,6,7].

Despite these findings, a significant research gap persists. Previous studies have predominantly relied on older models (GPT-4 and earlier) or free versions with restrictive context windows, failing to test the “reasoning” capabilities of the 2025 generation [8]. Furthermore, a critical gap remains regarding the “Anglocentric” bias of these models when applied to non-English data. Specifically, the performance of emerging models on non-English qualitative data remains largely unexplored. This study compared the latest OpenAI model, GPT-5, and Google’s Gemini 2.5 Pro (hereafter Gemini). Gemini was specifically chosen because of its free availability to students, which will likely lead to wider adoption in academic research [9].

This study addressed by 2 research questions. The primary research question addresses the methodological contribution of this study: “To what extent can GPT-5 and Gemini 2.5 Pro replicate human qualitative coding of sentiment in non-English health services data under model-optimized coding conditions?” The second, substantive question that guided codebook development was: “How has the sentiment toward nurses, as portrayed in local newspapers’ reporting, evolved throughout the COVID-19 pandemic (2020‐2023)?” The substantive question guided the development of the human codebook and the thematic structure of the analysis; the methodological question governs the study’s primary contribution and conclusions.

## Methods

### Research Design

To answer the research question on the evolution of sentiment toward nurses during the COVID-19 pandemic, this study used a qualitative research design rooted in comparative content analysis. This research deviates from traditional content analysis by incorporating a methodological benchmarking of human coding against AI.

The study follows a hybrid workflow. First, an inductive coding structure is established through manual human analysis to capture the nuances of the Dutch health care context. The analysis focused on identifying sentiment-related themes in the portrayal of nurses, including public appreciation, professional strain, and systemic critique, as expressed across the 4-year pandemic period. Second, LLMs are used to replicate this process. This dual approach allows for a critical assessment of the feasibility and reliability of using AI agents for qualitative sentiment analysis on health care–related data.

The following sections describe the data collection process, the human coding procedure, the AI analysis, and the validation procedures used to assess reliability.

### Data Collection

The data for this study consist of newspaper articles from local newspaper *De Limburger*, retrieved from the Nexis Uni database. As the leading regional newspaper in the province of Limburg, it provides a localized perspective on the health care crisis, specifically regarding hospitals and nursing staff in the region. The final dataset consists of n=317 articles, totaling 275,878 words (including headers and footers). The dataset was originally retrieved for a research project regarding the general valuation of nurses and has been adapted to answer the specific research question of this study.

The data collection spanned from January 1, 2020, to December 31, 2023. This timeline was specifically selected to encompass the entire trajectory of the COVID-19 pandemic in the Netherlands (specifically Limburg). It covers the start of the crisis (with the first confirmed Dutch case in February 2020), the period of active government mandates, the discontinuation of the national measures in May 2022, and extends through 2023 to capture retrospective articles reflecting on the pandemic era and reporting on the postpandemic situation [10].

To compile the dataset, a targeted search strategy was used. The geographic and institutional context was specified by the specific names of all hospitals located within the province of Limburg. For the target professional groups, keywords, including “Nurse” (verpleegkundige), “Health care worker” (zorgmedewerker), and related synonyms were used. For the pandemic context, the timeline was set, and keywords such as “COVID*,” “corona,” or “pandemic” (pandemie) were used.

**Figure 1.**
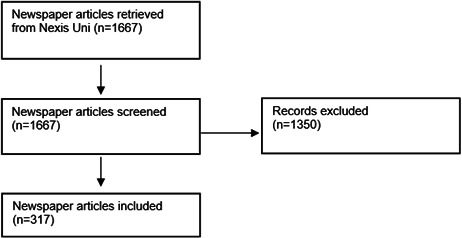
Newspaper article selection flowchart

For the initial retrieval, articles underwent a manual screening process to ensure relevance. Inclusion criteria required that articles be written in Dutch (avoid excessive dialect) and explicitly discuss nurses or health care workers in the context of the pandemic. Exclusion criteria were applied to remove articles that focused solely on epidemiological statistics (eg, infection rates) without qualitative discussion of staff, or articles that mentioned hospitals generally without specific reference to the nursing workforce. The full selection process, including the number of records excluded at each stage, is visualized in Figure 1.

### Phase 1: Code Generation

#### Human Qualitative Analysis

The human baseline was established through a rigorous collaborative protocol. The full dataset of 317 articles was coded by 2 independent researchers (BvdB and FCvdB) [11]. The codebook development was an iterative, consensus-driven process: the researchers held regular consultations to discuss coding discrepancies, resolve interpretive ambiguities, and refine thematic labels. Codes were only included in the final codebook once full consensus between the researchers was reached, ensuring the framework accurately reflected the nuances of the Dutch health care context. This iterative process of intersubjective agreement served to mitigate individual researcher bias and enhance the validity of the human baseline used for the subsequent AI comparison. Although the dataset consisted of Dutch articles, the human coding process was conducted in English, to enable the immediate creation of fitting codes, rather than maintaining 2 sets of codes and translating them post hoc.

The human analysis proceeded in 3 iterative steps. First, during open coding, the researcher manually assigned descriptive labels to phrases, paragraphs, or sections that reflected specific segments. In some cases, individual sentences were labeled with multiple codes. For example, the sentence: “Een angstaanjagende crisis leerde ons dat de ware helden in het ziekenhuis of het verpleeghuis werken (A terrifying crisis taught us that the real heroes work in hospitals or nursing homes.)*”* would get the code health care workers are heroes.

Second, axial coding was applied to group these open codes based on conceptual similarity. Through this manual synthesis, the researcher, for example, distilled “External support” as a distinct category. This category emerged through the aggregation of open codes related to signs of public appreciation, for example; health care workers are heroes, and nurses are supported by society.

In the third and final step, selective coding was used to integrate these categories into the overarching third-order themes presented in the results. For instance, “external support” and “internal improvements” both axial codes mainly prevalent in the first phase of the pandemic, together formed the third-order theme “Initial Praise.”

#### AI-Assisted Coding Strategy

To generate a comparative coding tree using AI, a structured prompting strategy was used. As established in recent methodological research, the quality of LLM output is heavily dependent on the precision of the prompt design [12]. Therefore, this study adopted a systematic workflow that builds upon the findings of Li et al [4]. Data processing and AI-assisted coding were conducted using the consumer-facing web interfaces of OpenAI’s ChatGPT (running the GPT-5 model) and Google (running the Gemini 2.5 Pro model). To mitigate the risk of algorithmic bias stemming from personalized memory or custom system instructions, both models were accessed using newly created, blank accounts with zero prior interaction history. Access to these platforms occurred between October 1, 2025, and November 28, 2025. Because the study used the standard conversational web interfaces rather than programmable API endpoints, specific technical parameters such as temperature, top-p sampling, and exact subversion identifiers (snapshots) were dictated by the platforms’ default settings at the time of access. To ensure optimal context retention within these constraints, each analytical phase (eg, codebook optimization and the coding of the subsample) was conducted within a single, continuous chat session per model.

#### Adoption of the Protocol

Whereas Li et al [4] used a direct, noniterative prompting strategy for their AI analysis, this study introduced a methodological modification to enhance validity; a self-optimization phase. Before the actual analysis began, both models were first tasked with rewriting the researcher’s draft prompt. Rather than using a fixed prompt for both models, this study opted for model-optimized prompts to test each model under its most favorable conditions. This approach is supported by recent research indicating that LLMs outperform on complex coding tasks when prompts are dynamically refined to align with the model’s internal latent space and processing logic. Furthermore, this iterative collaboration between researcher and AI aligns with the concept of using AI as a “co-researcher” [13,14].

#### Execution Procedure

The analysis was executed with a 6-step workflow for both GPT-5 and Gemini. This process ensured consistency between the 2 models. The specific steps are detailed in Table 1 and the full AI prompts are in S1 in Multimedia Appendix 1 for GPT-5 and S2 in Multimedia Appendix 1 for Gemini.

**Table 1.** AI prompting and analysis process.

Steps	Action	Description
1	Prompt optimization	The model was presented with the draft prompt and instructed to “Optimize this prompt for qualitative analysis by AI.”
2	Data ingestion	A new chat session was initiated. The dataset (275,878 words) was uploaded, and the model-optimized prompt was pasted.
3	Refinement	The model was instructed to “Look back at the data, see if you need to refine your codes, and create a table for the codes.” This forced a re-evaluation of initial findings.
4	Translation	The model was instructed to translate the final table into English for reporting consistency.
5	Codebook generation	The model was asked to generate a codebook for second-order axial codes, providing descriptions and interpretive guidelines.
6	Extraction	The final tables and codebooks were extracted for comparison.

### Phase 2: Code Application

To explore the potential of LLMs to serve as automated coding assistants, a second analytical phase was conducted. In this phase, the AI models were tasked with deductively applying the human-generated coding scheme to a subset of the data.

The objective was to qualitatively examine whether the AI agents could accurately process and apply the human-defined codes and to identify specific areas of divergence in interpretation.

#### Sampling Strategy

While Phase 1 involved ingesting the full corpus (275,878 words) for theme generation, Phase 2 applied codes to a 10% stratified random subsample (n=31 articles). This 2-phase design reflects the practical constraints of iterative AI coding and mirrors standard intercoder reliability (ICR) procedures in qualitative research. In qualitative content analysis, a 10% subsample is widely recognized as a pragmatic threshold to balance representative validation with the logistical demands of manual coding. Furthermore, as Krippendorff [15] emphasizes, reliability subsamples must be sufficiently large to capture the inherent variation of the data, and this 10% proportion serves as a robust benchmark for comparative assessments [16]. Rather than a purely randomized approach across the entire timeline, a 2-stage sampling method was used that targeted 15 chronological clusters, ultimately selecting articles from 3 distinct pandemic phases: September-November 2020 (initial crisis response), September-October 2021 (midpandemic sustainability), and the full year 2023 (postpandemic reflection). This stratified design was specifically chosen to facilitate a comparative analysis of coding reliability across different pandemic stages. The final sample consisted of articles from September 26, 2020, to November 18, 2020 (n=10); September 3, 2021-October 18, 2021 (n=11); and January 1, 2023-December 31, 2023 (n=10).

#### Reliability and Validation

To validate the human coding baseline used for comparison, an independent ICR assessment was conducted on the Phase 2 subsample (n=31 articles). A second researcher (FCvdB) independently coded this subsample using the final, consensus-driven codebook developed in Phase 1. ICR was assessed only for this deductive coding phase using the intercoder agreement tool in ATLAS.ti (version 25; ATLAS.ti Scientific Software Development GmbH). All coders independently applied the predefined codebook to the same set of 31 newspaper articles. The unit of analysis was the coded quotation (ie, a meaningful text segment), with coders assigning one or more codes to any passage they considered consistent with the code definitions. Multiple instances of the same code could be assigned within a single article when distinct passages represented separate occurrences of the concept. Following independent coding, the coding projects were merged in ATLAS.ti. Using its default quotation overlap settings, the software identified overlapping coded quotations across coders and calculated Krippendorff alpha—a robust measure for qualitative content analysis [16]—using its built-in intercoder agreement tool. Agreement between the primary and second human coder yielded a Krippendorff α of 0.703, indicating acceptable agreement [17]. To quantify the interpretive divergence between human and AI coding, the same procedure was used to calculate Krippendorff alpha for the primary human researcher versus GPT-5 and Gemini.

#### Iterative Refinement of the AI Process

The execution of the AI coding involved an iterative process consisting of 2 rounds. This approach was chosen because the initial round revealed that the AI produced outputs inconsistent with the human codebook without additional context, revealing the necessity for methodological improvements.

##### Round 1: Process Pilot

In the first round, a procedure was tested using GPT-5, in which the 10% sample was uploaded with the instruction to code full sentences based strictly on the human codebook (which was also uploaded to the AI). Despite using an optimized prompt as in Phase 1, the pilot exposed 2 critical limitations in the model’s performance.

Firstly, the model generated contextually misaligned codes, applying human codes too broadly. Specifically, codes such as “Internal Support” were incorrectly assigned to non–health care contexts, such as the hospitality sector, thereby deviating from the research question’s specific focus on nursing. Furthermore, data omission proved to be a technical hurdle. When processing larger files or longer articles, GPT-5 frequently omitted relevant paragraphs, resulting in incomplete coding coverage.

##### Round 2 Optimized Step-by-Step Protocol

To mitigate the issues from round one, the protocol underwent substantial refinement for the final analysis. The human codebook was rigorously revised to provide more context and tighten up the definitions by giving examples (S5 in Multimedia Appendix 1). To bridge the gap between human intent and AI workflows, this revised codebook was uploaded to the respective AI models to generate an optimized version, as provided in S6 in Multimedia Appendix 1 (GPT-5) and G (Gemini).

Additionally, the operational workflow was changed from an all-at-once approach to an article-by-article approach. Each article was processed individually by uploading its text to prevent data omission. These changes resulted in the approach described in Table 2.

**Table 2.** Prompting strategy for AI-assisted code application.

Steps	Action	Description
1	Codebook optimization	The revised codebook was uploaded to the AI model. The model was then asked to optimize it for AI use of qualitative coding.
2	Prompt optimization	The model was presented with the draft prompt and instructed to “Optimize this prompt for qualitative analysis by AI.”
3	Data ingestion	A new chat session was initiated where the 10% dataset was uploaded, as well as the AI-optimized codebooks.
4	Coding	The article’s text was given to the AI model with the optimized prompt to code the sentences. After the coding for that article finished, it was asked to refine the coding. This process was repeated for each article.
5	Extraction	The codes were transferred manually to ATLAS.ti (ATLAS.ti Scientific Software Development GmbH) to create a fully coded file with AI-assisted coding. To ensure data integrity during the manual transfer of AI-generated codes into ATLAS.ti, a systematic cross-verification process was performed against the raw AI outputs.

### Analysis of the Output

The resulting AI-coded data were extracted and qualitatively compared to the human coding of the same articles. This comparison allowed for an assessment of the interpretative alignment between the AI agents and the human researcher, identifying where the AI models differed from each other and the human coding.

Because GPT-5 optimized the code names for its internal processing logic during the prompt optimization step (eg, converting “Care takes a toll” to “CARE_TOLL”), a manual mapping procedure was required. All AI-applied codes were systematically mapped back to their original human codebook categories prior to calculating the frequencies presented in Table 3. This mapping was verified against the detailed descriptions in the AI-optimized codebooks to ensure that the quantitative comparison reflected true semantic alignment rather than superficial label matching.

**Table 3.** Difference in codes used.

Code	GPT-5, n (%)^a^	Gemini, n (%)^a^	Human analysis, n (%)^a^
Better internal conditions	28 (15.47)	29 (15.85)	11 (7.97)
Care takes a toll	15 (8.29)	4 (2.19)	6 (4.35)
Disappointment in Government	11 (6.08)	17 (9.29)	12 (8.70)
Discrepancy between society and health care workers	14 (7.73)	25 (13.66)	12 (8.70)
External support	18 (9.94)	21 (11.48)	19 (13.77)
Issues around vaccination	7 (3.87)	13 (7.10)	7 (5.07)
Nurses are (emotionally) exhausted	38 (20.99)	27 (14.75)	16 (11.59)
Problem-solving	20 (11.05)	23 (12.57)	25 (18.12)
The great resignation	19 (10.05)	14 (7.65)	20 (14.49)
Unease	11 (6.08)	10 (5.46)	10 (7.25)
Total code applications	181	183	138
Quotations (multiple codes per quotation possible)	176	178	136

a Percentages are based on the total number of code applications within each coder.

### Ethical Considerations

This study received ethics approval from the Faculty of Health, Medicine and Life Sciences Research Ethics Committee of Maastricht University (FHML-REC/2020/110). Because the research involved processing copyrighted newspaper articles from *De Limburger* via the Nexis Uni database through proprietary AI models hosted by OpenAI and Google, data governance was carefully handled. All text processing was conducted strictly for analytical purposes under academic research exemptions. We recognize that using commercial LLMs via standard web interfaces introduces distinct risks, such as data retention, privacy concerns, and proprietary model bias compared to enterprise API solutions that offer zero data retention guarantees. Consequently, our team navigated the evolving legal landscape surrounding AI data ingestion and intellectual property to ensure strict adherence to institutional data governance policies throughout the analytical process.

## Results

### Phase 1: Code Generation

The prompts and analyses yielded 3 coding trees, corresponding to the human coding, GPT-5, and Gemini. Table 4 presents the third-order themes derived from each analysis. A detailed breakdown for the first- and second-order codes is provided in S1 in Multimedia Appendix 1 for GPT-5, S2 in Multimedia Appendix 1 for Gemini, and S3 in Multimedia Appendix 1 for the human coding.

**Table 4.** Comparing the bigger themes.

Themes	Human coding	GPT-5	Gemini
Theme 1	Initial praise (external support and internal improvements)	A. From “heroes” to honored professionals (2020)	Unanimous hero worship and public solidarity (code appears in 2020)
Theme 2	Arising problems and frictions (shift in society and recognition)	B. Strain, burnout, and workforce erosion (2020‐2022) C. Recognition politics and contested compensation (2022‐2023)	From applause to activism (code appears around 2020‐2021)
Theme 3	The new crisis (nursing shortages and how to deal with it)	D. Transition to the “new normal” and fading crisis salience (2023)	Structural crisis and professional reorientation (code appears around 2022‐2023)

### Theme 1

Theme 1 demonstrates a high degree of similarity across all 3 analyses. Both AI models identified “Hero” as a distinct, standalone theme. Although the human analysis grounded this concept within a broader category regarding working conditions, the underlying findings are nearly identical. This is evidenced by the human open code “Health care workers are heroes,” which appeared frequently (n=47). Therefore, despite the slight structural difference in grouping, the “Hero” narrative represents a shared and comparable primary finding across all 3 methods.

Notably, the human analysis offered a more multifaceted perspective. The human analysis, the first theme “Initial praise,” combined external support (including the “Hero” theme) with improvement of internal working conditions. Codes related to improvements in internal working conditions were absent in the open codes of both Gemini and GPT-5. They focused solely on the external support and praise. Nevertheless, all 3 analyses converged on a similar positive sentiment regarding the start of the COVID-19 crisis in 2020.

### Theme 2 (Coded by GPT-5 as B Strain and C Recognition)

Theme 2 showed a strong agreement across all 3 analyses regarding the problems reported in the data. Despite structural differences in how the AI model organized these findings, specifically GPT-5 splitting the topic into 2 parts, the underlying content is aligned with the human analysis.

#### Gemini

Although Gemini labeled its theme with the narrative title “From Applause to Activism,” an examination of the first- and second-order codes reveals it covers the same problematic aspects as the human coding. Specifically, codes such as “2.1: Physical and Mental Exhaustion (The Toll)” and “2.2: System Failure and Structural Shortages” directly align with the societal challenges and lack of appreciation identified in the human analysis.

#### GPT-5

The comparison with GPT-5 is more complex structurally, as the model identified 4 total third-order themes. However, when combining themes B and C, they form the same spectrum as theme 2 from both the human analysis and Gemini. Theme B, “Strain, burnout, and workforce erosion (2020‐2022),*”* addresses the internal dimension: the direct toll on nurses, including exhaustion, feelings of undervaluation, and resulting strikes. Theme C, *“*Recognition politics and contested compensation (2022‐2023),” focuses on the external dimension: Political conflicts, the role of unions, and the decline of governmental support. In summary, while GPT-5 creates a distinction between internal strain and external politics, the aggregate of these themes mirrors the “Problem” theme of both the Gemini and human datasets.

### Theme 3

Across all 3 analyses, the final theme revolved around the emergence of a new situation following the peak of the pandemic. However, this theme showed the greatest divergence between the methods.

The human analysis and Gemini both conceptualized their final themes as a “New Crisis.” They identified a direct link between the pandemic and the subsequent fallout, explicitly incorporating codes regarding nurse exodus (The Exit) and structural staff shortages. For these 2 methods, the end of the pandemic was defined by the substantive new problems that emerged.

In contrast, GPT-5 conceptualized the final theme as “Transition to the New Normal.” Rather than focusing on the substantive issues (like nurse shortages), GPT-5 adopted a meta-perspective; it focused on the fading frequency of news reporting and the changing tone of *De Limburger*. By generating 4 themes instead of 3, GPT-5 structurally separated the manner of reporting from the content of the reports. It isolated the “exodus and shortages” in Theme B (internal strain), whereas Gemini and the human analysis viewed these elements as intrinsically interconnected with the postpandemic reality. Consequently, GPT-5 saw a “fading of attention,” and Gemini analysis saw the “start of a crisis.”

### Codebook

Both AI models were instructed to generate a codebook describing their axial codes. This step is essential for qualitative rigor, as a codebook clarifies the definitions of codes and allows for the assessment of intercoder reliability. GPT produced a descriptive, narrative-style codebook (see S1 in Multimedia Appendix 1). It included an “Analytic Description” (explaining the core concept of the code) and an “Interpretive Note” (guiding the researcher on how to read the code). While rich in context, it was less structured for quick referencing.

Gemini provided a highly structured codebook designed for practical application (see S2 in Multimedia Appendix 1). It used columns for description and “Interpretation/Coding Rules.” Notably, Gemini enhanced the utility of its codebook by providing concrete examples and direct citations to the original articles, thereby increasing transparency and verifiability.

### Phase 2: Code Application

To further examine how the LLMs applied the coding scheme, a 2-round approach was performed. This section details the process improvements and compares the AI output with the human baseline. Starting with the results of Round 1 with only GPT-5 and going more in depth for the second, where both AI models were represented.

#### Round 1 (GPT-5 Only)

The first round was conducted exclusively to test the coding procedure. This pilot revealed several technical limitations. First, the model produced hallucinations, including sentences in its output that did not correspond with the uploaded text. For instance, GPT-5 generated the phrase: “We willen ook de andere zorg overeind houden” while the original text was: “Alle ziekenhuizen samen willen de reguliere, niet-corona-zorg zo lang mogelijk overeind houden” or “nu wil je ook de andere zorg overeind houden.” Although the meaning is similar, the lack of accuracy is problematic for analysis.

Furthermore, the model had poor organizational consistency. It provided the articles out of chronological order and repeatedly halted output, requiring user input to continue. Additionally, data omission occurred; the model failed to code entire articles or skipped random sections, especially toward the end of longer newspaper articles.

#### Round 2 GPT-5

Based on the lessons from Round 1, a refined protocol was applied to both GPT-5 and Gemini. This included a step-by-step approach and the introduction of a confidence score to filter the results. It should be noted that these confidence scores are uncalibrated outputs derived from the AI’s internal logic rather than calibrated probability estimates. Consequently, they reflect the unique prompting and processing differences between the models and should not be treated as objective evidence of coding quality or absolute accuracy.

In the second round, the output structure was improved. However, during the optimization process, GPT-5 applied different code names than those provided in the input, although the descriptions remained consistent. The codes “Better internal working conditions” and “Care takes a toll” proved the most difficult for the model to apply correctly. This resulted in sentences being coded that were unrelated to the code’s definition. Additionally, “Nurses are (emotionally) exhausted” was applied more broadly than intended. To mitigate this, the model was instructed “Refine the codes; not every sentence needs a code, only code when you think it is necessary.” Following this instruction, only coded sentences with a confidence score of ≥0.80 appeared. Therefore, the analysis was restarted for the articles before this prompt. Table 5 presents the output for the first article. The original table also created notes about why it coded this sentence, but these are taken out of Table 5 because Gemini did not provide notes and the notes were summaries of the codebook.

**Table 5.** GPT-5 output for the first article.

Sentence	Applied axial codes	Confidence
“Tijdens de eerste golf waren het vooral de afdelingen voor intensieve zorg die werden overspoeld. Nu zijn het vooral de verpleegafdelingen die volstromen.” (“During the first wave, it was mainly the intensive care units that were overwhelmed. Now it’s mainly the general wards that are filling up.”)	CARE_TOLL	0.85
“Die mensen moeten we wel al isoleren, wat extra belastend is voor het personeel.” (“We have to isolate those patients, which puts an extra strain on the staff.”)	EXHAUSTION	0.82
“Het personeelstekort in de zorg is alleen maar groter geworden nu ook veel personeel zelf besmet raakt met corona of uitvalt in afwachting van een coronatest.” (“The staffing shortage in healthcare has only gotten worse now that many staff members are contracting COVID-19 themselves or are sidelined while awaiting a COVID-19 test.”)	RESIGNATION	0.88
“Een verpleegkundige opleiding duurt vier jaar. Dat is niet voor niets.” (“Nursing school takes four years. There’s a good reason for that.”)	PROBLEM_SOLVING	0.75
“Zo worden bijvoorbeeld gepensioneerden weer opgeroepen.” (“For example, retirees are being called back into service.”)	EXTERNAL_SUPPORT	0.83
“Er wordt overal aan personeel getrokken.” (“Staff are being pulled from everywhere.”)	RESIGNATION	0.80

The codes “Better internal working conditions” and “care takes a toll” were the least accurate with the improved codebook and, therefore, resulted in some sentences being coded that had nothing to do with the meaning of the code. Also, “nurses are emotionally exhausted” was applied more broadly than intended on some occasions. Later, during the coding with GPT-5, it was instructed that “Refine the codes; not every sentence needs a code, only code when you think it is necessary.” This resulted in it only showing sentences with a confidence score of 0.80 or higher for the remaining articles, and overcoding happened less. Therefore, the outputs before this comment were checked and reassessed with GPT-5 by starting over, and all previous codes, including those with a score lower than 0.80, were removed from the ATLAS.ti file (the highest confidence score was 0.97; GPT-5 ended with 176 quotations). Table 5 presents the raw, unfiltered output for the first article (including a score of 0.75) to illustrate the model’s baseline behavior before the manual application of the 0.80 threshold.”

#### Round 2 Gemini

A preliminary round was not conducted for Gemini, as the methodological insights from the GPT-5 pilot (The codebook, providing more context, and the article-by-article approach) were directly transferable to Gemini. Gemini began by optimizing the codebook and prompt, including the request for confidence scores. Compared to GPT-5, Gemini provided less conversational text; its outputs were direct and strictly followed the requested table format, while GPT-5 provided notes and sentences underneath the table stating its reasoning. Regarding the confidence scores, Gemini generated high confidence scores, predominantly assigning scores of 0.90 or 1.00, and applying a score lower than 0.90 (specifically 0.80) only once. Because Gemini consistently provided output scores at or above the 0.80 threshold from the beginning, without generating quotations that were clearly divergent from the codebook, it did not undergo the same mid-process prompt reminder (“Refine the codes.") or manual filtering applied to GPT-5. This can be seen as a procedural asymmetry between the 2 models, which arose dynamically from their differing baseline responses. The highest confidence score for GPT-5 was 0.97, with scores frequently falling between 0.80 and 0.90. Table 6 presents the output for the first article (the same as for GPT-5).

**Table 6.** Gemini output for the first article.

Sentence (verbatim)	Applied axial codes	Confidence
“Die mensen moeten we wel al isoleren, wat extra belastend is voor het personeel,” zegt Peeters. (“We have to isolate those people anyway, which puts an extra strain on the staff,” “We have to isolate those people anyway, which puts an extra strain on the staff,” says Peeters.)	Nurses are (emotionally) exhausted	0.90
“Het personeelstekort in de zorg is alleen maar groter geworden nu ook veel personeel zelf besmet raakt met corona of uitvalt in afwachting van een coronatest.” ( *“*The staffing shortage in healthcare has only gotten worse now that many staff members are also becoming infected with COVID-19 or are out of work while awaiting a COVID-19 test.”)	The great resignation	1.00
“En er wordt overal aan personeel getrokken.” (“And staff are being pulled in from all over.”)	Problem-solving	0.90
“Zo worden bijvoorbeeld gepensioneerden weer opgeroepen.” ( “For example, retirees are being called back into service.”)	Problem-solving	1.00

### Quantitative Comparison of Coding Frequencies

To compare the performance of the AI models against the human baseline, both the total frequency of code application and formal ICR were calculated. The human baseline was validated through an independent double-coding process, resulting in a Krippendorff α of 0.703, indicating acceptable intersubjective agreement. Table 3 presents the coding frequencies and the corresponding reliability scores (α) for the AI models when compared to the human baseline. As shown in Table 3, both AI models generated a substantially higher volume of code applications than the human analysis (181 and 183 vs 138). The calculated reliability scores (α human-GPT5=0.487; α human-Gemini=0.507) quantify the interpretive divergence between the human baseline and the AI agents.

A possible explanation is that the researcher coded more sentences under the same quote; this would count as one quotation instead of multiple quotations by the AI model. Another explanation is that the AI models seemed to “ overcode,” likely identifying thematic relevance in sentences where the human was more conservative. Consequently, the AI models appear more sensitive to potential signals in the text, while the human coder applied stricter thresholds for assigning codes.

Specific divergences highlight different sensitivities between the models. For instance, both models used the code “Nurses are (emotionally) exhausted” substantially more compared to the human baseline (n=16), with GPT-5 applying it 38 times. Furthermore, individual outliers occurred: GPT-5 applied “Care takes a Toll” more than twice as much (n=15) compared to the human coding (n=6), while Gemini was an outlier for “Discrepancy between society and health care workers” (n=25), exceeding both GPT-5 (n=14) and the human analysis (n=12).

### Qualitative Analysis of Coding Errors

A qualitative review reveals specific errors shared by the models. Notably, both AIs incorrectly coded the sentence, “Het administratief allemaal verwerken, wordt daarna nog alle hens aan dek, zegt een woordvoerder van Zuyderland.” They applied the code “Nurses are (emotionally) exhausted,” even though the sentence referred to administrative measures, not nurses. Similarly, the phrase “Dat beleid hanteren we in ons ziekenhuis” was coded as “Better internal conditions” by both models despite the context being explicitly not an improvement. Both models individually coded a sentence a human might not code, but only shared these 2.

Finally, a difference in adaptability was observed. GPT-5 made significant changes when asked to refine its coding based on feedback. In contrast, Gemini exhibited less variation in output, typically making few to no changes after a refinement prompt.

## Discussion

### Principal Findings

This discussion presents the key findings from the comparison of human and AI-driven qualitative analysis, followed by methodological insights gained during the process. It concludes with an overview of the study’s limitations and directions for future research.

### Thematic Comparison

The first phase of this analysis revealed that while AI and humans identify similar broad patterns, their underlying analytical logic differs in meaningful ways. For the first theme, both AI models independently identified the emergence of a “Health care heroes” narrative, a finding that was deliberately excluded from the human analysis. This exclusion was a conscious methodological choice, having previously analyzed this dataset in a related research project on nurse valuation, the researcher deprioritized the heroes framing in favor of different themes. In retrospect, this decision illustrates a broader risk in qualitative research that expertise and prior exposure can introduce blind spots. The AI’s inclusion of this theme, free from prior coding frameworks, paradoxically served as a corrective, confirming that the theme was present in the data.

However, this finding also warrants a degree of caution. The human coding framework was developed inductively from the newspaper corpus, but the researcher had prior exposure to related interview data from the same broader research program on nurse valuation. It is therefore possible that certain codes, particularly those capturing external appreciation and internal working conditions, were implicitly shaped by that earlier analytical work rather than emerging purely from the newspaper data alone. This form of researcher positionality was partially managed through iterative consultation with a second researcher (FCvdB), but cannot be entirely ruled out as a source of framing bias. Notably, the AI models, having no access to this prior work, independently converged on several of the same themes, which offers some reassurance that the core findings reflect genuine patterns in the data rather than artifacts of the human coding frame.

A critical quantitative finding (Table 3) was the volume of coding; both AI models identified substantially more quotations than the human researcher (approximately 180 vs 136). It is important to contextualize this difference, however, in light of the study’s 2-phase design. While Phase 1 involved ingesting the full 275,878-word corpus for theme generation, Phase 2 applied codes to a 10% stratified random subsample of 31 articles. Formal ICR assessments confirmed an interpretive divergence, with Krippendorff α scores of 0.487 for GPT-5 and 0.507 for Gemini, compared to a human consensus baseline of α=0.703. The overcoding observed in Phase 2, therefore, seems to reflect AI behavior under deductive coding conditions, applying a predefined human codebook to new material rather than the models’ free inductive behavior in Phase 1. The overcoding may partly reflect the models’ difficulty in calibrating the threshold for code application, prioritizing semantic presence over the interpretive significance applied by human coders. Whereas human coders emphasize contextual coherence and interpretive significance when defining meaningful units of text, LLMs appear to default to identifying each semantically relevant text fragment separately, resulting in a finer-grained coding approach. As such, lower Krippendorff alpha scores for GPT-5 and Gemini may stem more from quotation unitization differences rather than genuine disagreement.

The quantitative difference in code frequencies (Table 3) raises the question: do the AI models “overcode” or do the human researchers “undercode”? By validating the human baseline through ICR (α=0.703) and consensus for interpretation, a robust benchmark was established. Against this human baseline, the AI models have a higher number of code quotations, suggesting a lower threshold for identifying semantically relevant text. As noted by Wachinger et al [5], AI models demonstrate high proficiency at identifying descriptive themes based on semantic patterns but exhibit lower accuracy with interpretive weight. The results of this study extend this finding by identifying a potential structural dichotomy: AI models appear to code for semantic presence (does this concept appear in the sentence?), whereas human researchers appear to code for interpretive significance (does this concept meaningfully drive the narrative?). Consequently, LLMs currently function as high-sensitivity “Content Retrievers” rather than nuanced “Context Interpreters.” They successfully detect textual signals but require human oversight to assign appropriate interpretive weight to those findings. This distinction reinforces the assertion by Kitamura et al [3] that, despite reasoning advancements, LLMs remain pattern-matching engines that require human oversight to assign appropriate weight to findings.

### Model Comparison: GPT-5 vs Gemini 2.5 Pro

When comparing the specific performance of the models, distinct behavioral profiles emerged that led to different recommendations for academic use. GPT-5 provided extensive narrative descriptions and longer responses, often including codes with lower confidence scores. Conversely, Gemini was notably more concise, adhering more strictly to the codebook and assigning high confidence scores.

Within the specific parameters of this study, Gemini demonstrated characteristics that may be advantageous for structured qualitative work. However, given the differing optimization prompts, the sequential workflow, and the uncalibrated confidence scores, this observation requires further controlled testing rather than a definitive recommendation. Gemini enhanced the utility of its codebook by providing concrete examples and direct citations to the original articles, thereby increasing transparency and verifiability. This distinction is critical when considering the “hallucination vs omission” dichotomy described by Li et al [4]. While GPT-5’s sensitivity captures relevant nuance, it also introduces more “noise.”

### Methodological Insights for AI-Assisted Coding

The first round of coding yielded suboptimal results. GPT-5 required more context. Providing only 10% of the data and a codebook with the axial codes resulted in GPT-5 coding irrelevant data and coding too much, whereby the overall quality of the coding was deemed poor. Furthermore, the process of coding with a chat interface and then transferring the data by hand to ATLAS could be considered a tedious process that took more time than doing the coding by hand. This suggests that while AI models are useful tools to use for the qualitative coding process, they require substantial contextual information and context to code precisely, and even then, sometimes unexplainable sentences get coded.

The need for iteration was further highlighted by the variance in how the models processed instructions. For example, in the second round, GPT-5 returned its results using its own code names (eg, CARE_TOLL) instead of the exact names provided in the codebook. While the meanings were consistent, this “interpretive friction” illustrates that the AI is not a static tool but a dynamic processing variable in the process, requiring researchers to be vigilant in managing and validating its output. This necessitates a “Human-in-the-Loop” workflow, a concept reinforced by Cook et al [17] who emphasize that AI tools are “probabilistic pattern-matchers” requiring expert intervention to prevent unsophisticated analyses. Furthermore, effective human-in-the-loop use also requires critical thinking by the user [18]. Because AI models can produce highly fluent but occasionally flawed outputs, researchers cannot accept the output at face value. Instead, AI codes must consistently be verified against the data, and researchers must be mindful of limitations from the model in understanding the deep context [18].

To get the best results and make sure the AI model refrains from skipping articles, going for an article-by-article approach while confirming if it has all the codes for that article yielded higher reliability. In the first round, the approach of asking to do all the articles at once exceeded the model’s effective context processing capacity, and this resulted in hallucinations, GPT-5 skipped articles, and coding in a random order.

### Limitations

Several limitations should be noted. First, researcher bias cannot be ruled out; the dataset had been previously coded by the researchers, potentially influencing the human baseline. Furthermore, the human analysis may have endured fatigue compared to the consistent computational processing of the AI.

Second, the prompting and coding procedures were not designed as a controlled, head-to-head benchmark between the 2 models. Because the models were allowed to optimize their own prompts and codebooks, they ultimately operated under different effective instructions. Furthermore, in Phase 2, GPT-5 was piloted first. Insights and corrections derived from its initial failures were subsequently transferred to the Gemini workflow, which may have provided Gemini with an unintended sequential advantage. This design limitation means the results should be viewed as an exploration of each model’s independently optimized capabilities rather than a standardized comparative trial.

Third, the study was conducted using consumer-facing web interfaces rather than programmable API calls. Consequently, critical technical parameters necessary for strict reproducibility, such as exact model version identifiers, temperature, sampling parameters, system prompt configurations, and session management details, were neither standardized nor recorded. The conversational workflow means other researchers using GPT-5 or Gemini 2.5 Pro may interact with different underlying model snapshots or dynamic updates. For transparency, the web interfaces for both models were accessed between October 1, 2025, and November 28, 2025. To ensure strict reproducibility, future methodological benchmarking must use API-based workflows. Integrating an API pipeline would lock in specific model versions, control sampling parameters, and simultaneously eliminate the potential for human error associated with the manual transfer of AI-generated codes into ATLAS.ti. As Okuyama et al [6] note, balancing speed and accuracy requires integrated workflows; the tedious manual entry in this study negates some of the efficiency gains promised by AI.

Fourth, the analysis involved a cross-lingual process: the source corpus consisted of Dutch newspaper articles, while both the human coding and AI prompting were conducted in English. This design choice was implemented to maintain a unified analytical framework and to ensure consistency between human and AI coding, thereby avoiding the complexity of maintaining and translating multiple codebooks. However, this introduces an interpretive layer where culturally or linguistically specific expressions may be attenuated.

Finally, AI is rapidly improving, and models are continuously updated. During the writing process of this study, new models, GPT-5.1 and Gemini 3, have been released [19,20]. The specific performance metrics of GPT-5 and Gemini 2.5 Pro that are used in this study may soon be superseded. However, this limitation paradoxically underscores the relevance of this study. If current models already demonstrate the capability to perform comparable qualitative analysis, as evidenced by the high thematic alignment, it is reasonable to hypothesize that future iterations, trained on more data, will only improve in precision and reliability. Therefore, this study serves as a conservative baseline for the potential of AI in qualitative research.

### Ethical Implications

A final methodological and ethical consideration involves data governance. This study processed copyrighted newspaper articles from the Nexis Uni database through proprietary AI models hosted by OpenAI and Google. While this was done strictly for analytical purposes under academic research exemptions, the use of “black box” commercial LLMs introduces risks regarding data retention, privacy, and proprietary model bias. Researchers must navigate an evolving legal landscape regarding intellectual property and AI ingestion. Future studies using AI-assisted qualitative analysis must ensure strict adherence to institutional data governance policies, particularly when using commercial web interfaces rather than enterprise API solutions with zero data–retention guarantees.

### Future Research

Overall, Gemini, as well as GPT-5, processed the Dutch data without observable performance limitations, and the coding was done in English. The LLMs did not accurately process jargon abbreviations like “OK” (Operational Theater), which, for most Dutch persons, is easy to recognize in the context of health care data. This is in line with previous research stating that AI demonstrates lower sensitivity to non-English nuances [8]. Therefore, if in the future Dutch-trained AI becomes available, future research could test Dutch-trained LLMs.

### Conclusion

This research indicates that LLMs like GPT-5 and Gemini are not simple replacements for qualitative researchers but rather complex assistants that possess a clear duality. The findings suggest that the models demonstrate a strong capacity for identifying bigger themes within larger bodies of non-English data, even highlighting a prominent “heroes” theme that was deliberately omitted by the human researcher. This capability makes them suitable for initial thematic exploration and identifying potential blind spots.

Their success on the detailed analysis level is, however, more nuanced. When applying specific codes, the models coded significantly more frequently than the human consensus baseline, identifying nearly every semantic instance of a concept rather than filtering for narrative importance. Consequently, this study concludes that these discrepancies point toward a potential divergence in analytical logic; AI models appear to code for semantic presence, while human researchers prioritize interpretive significance.

Therefore, the primary contribution of this study is methodological. It demonstrates the difference between a one-shot prompt leading to hallucinations and unusable data on a detailed level. The findings emphasize that rigorous, iterative, and context-rich “human-in-the-loop” workflows are important for maximizing the reliability of AI tools in qualitative research. Ultimately, LLMs can be a valuable addition for qualitative researchers, but this study provides a transparent example of the methodological adaptation required to harness their strengths while mitigating their weaknesses.

## Supplementary material

10.2196/98374 Multimedia Appendix 1 Supplementary data: AI outputs and testing phases.

## Abbreviations

ICR: independent intercoder reliability
LLM: large language model
