# Supplementary material for: Analyzing Nurse Sentiment in a Dutch Regional Newspaper During the COVID-19 Pandemic: Comparative Content Analysis of GPT-5, Gemini 2.5 Pro, and Human Coding
Source: J Med Internet Res. 2026 Aug 13;28:e98374. doi: 10.2196/98374 (PMC13472621; doi:10.2196/98374)
Supplement: Supplementary file 1 [file jmir-v28-e98374-s001.docx]

# Appendix Article

## S1. GPT 5- Prompts and outcomes

### Coding tree

Initial prompt

*You are a researcher. I have uploaded 860 pages of newspaper data. You will do what is called qualitative coding. Create a coding tree that consists of 1. Open codes, group them in axial codes and group those in a few themes (3rd order codes). The codes should be able to be applied to themes that are visible in the text and revolve around the research question: How has the sentiment towards nurses, as portrayed in De Limburger’s reporting, evolved throughout the COVID‑19 pandemic (2020–2023)?*

Asked GPT 5 to optimize the prompt:
*Optimize this prompt: You are a researcher. I have uploaded 860 pages of newspaper data. You will do what is called qualitative coding. Create a coding tree that consists of 1. Open codes, group them in axial codes and group those in a few themes (3rd order codes). The codes should be able to be applied to themes that are visible in the text and revolve around the research question: How has the sentiment towards nurses, as portrayed in De Limburger’s reporting, evolved throughout the COVID‑19 pandemic (2020–2023)?*

Optimized prompt

***Optimized Prompt***

*You are a* ***qualitative researcher*** *conducting thematic analysis of a large newspaper corpus (860 pages from De Limburger).
Your task is to perform* ***qualitative coding*** *following the classic* ***three-level coding structure*** *used in the constant comparative method:*

1. ***Open Codes*** *– Identify specific, descriptive concepts visible in the text (e.g., expressions, sentiments, narratives, metaphors).*
2. ***Axial Codes*** *– Group related open codes into broader conceptual categories that capture relationships or shared meanings.*
3. ***Themes (3rd-order codes)*** *– Synthesize the axial codes into a small number of overarching themes that represent the main patterns in the data.*

*Focus your analysis on the* ***research question****:*

*How has the sentiment towards nurses, as portrayed in De Limburger’s reporting, evolved throughout the COVID-19 pandemic (2020–2023)?*

*When constructing the* ***coding tree****, ensure that:*

- *Codes reflect both* ***positive and negative sentiment shifts*** *over time.*
- *You note recurring* ***frames, language choices, metaphors****, or* ***story types*** *(e.g., heroism, burnout, public appreciation, blame).*
- *Each code is* ***clearly defined*** *and can be* ***applied systematically*** *to the dataset.*
- *The coding tree is* ***hierarchical, comprehensive, and non-overlapping****.*

*Present your output as a* ***structured coding tree****, formatted as follows:*

*Theme A: [Theme label]*

*Axial Code 1: [Category label]*

*- Open Code 1: [description]*

*- Open Code 2: [description]*

*Axial Code 2: ...*

*Theme B: ...*

*Include a brief* ***summary paragraph*** *explaining the rationale for the structure — why the themes were chosen and how they connect to the evolution of sentiment toward nurses.*

Look back at the data see if you need to refine your codes and create a table for the codes

...

Translate the table to English

| **Theme** | **Axial Code** | **Open Code** | **Definition / Description** | **Sentiment Polarity** | **Example / Typical Expression (translated)** |
| --- | --- | --- | --- | --- | --- |
| **A. From “heroes” to honored professionals (2020)** | Public gratitude & symbolic appreciation | “Care heroes” / applause rituals | Collective public admiration; symbolic hero language | Positive | “Applause for our care heroes” / “Nurses deserve more than applause” |
|  |  | Material gratitude (bonus, statues) | Calls for financial or material rewards as tokens of respect | Positive → neutral | “A care bonus as a token of appreciation” |
|  | Moral elevation of bedside work | Media emphasis on selflessness | Portrayal of nurses as moral exemplars and frontline saviors | Positive | “They put everything aside for their patients” |
|  |  | Local community gestures | Small-scale civic acts honoring nurses | Positive | “Local baker treats hospital staff to thank them” |
| **B. Strain, burnout & workforce erosion (2020–2022)** | Overload & emotional toll | Exhaustion and stress | Reports of overwork, emotional fatigue, sickness absence | Negative | “Nurses at their limit” / “Hospitals groaning under absenteeism” |
|  |  | “ICs of elderly care” | Nursing homes described as intensive-care-like → metaphor for burden | Negative | “Nursing homes as ICs without resources” |
|  | Exodus & retention crisis | Staff departures | Reports of nurses quitting due to workload/pay issues | Negative | “15% of ICU staff have left since COVID began” |
|  |  | Lack of new entrants | Concerns about unfilled training positions, long-term shortage | Negative | “No new staff entering care, only more leaving” |
|  | Labor contention & collective action | Strike / “Sunday service” actions | Organized protests or symbolic work stoppages | Neutral → negative toward system | “Hospitals operate on Sunday schedule in protest” |
|  |  | Insufficient government support | Frustration with inadequate policy measures | Negative | “A drop in the ocean” |
| **C. Recognition politics & contested compensation (2022–2023)** | Policy critique & governance gaps | Institutional neglect | Post-crisis evaluations exposing systemic failures | Negative | “Safety Board: a silent disaster in care homes” |
|  |  | Political accountability | Parliament or unions demanding redress | Neutral → negative | “Parliament demands action for sick healthcare workers” |
|  | Long-COVID recognition battles | Exclusion from compensation schemes | Disputes over narrow eligibility windows | Negative | “Compensation only for the first wave – unfair” |
|  |  | Union legal action | Unions using legal tools to enforce compensation | Neutral → negative (toward government) | “FNV union uses legal leverage against the cabinet” |
|  | Symbolic but uneven appreciation | Small gestures | Non-monetary acknowledgments post-pandemic | Mildly positive | “Free museum entry for healthcare staff” |
|  |  | Unresolved strain | Ongoing staff shortages and high workload | Negative | “Workload remains sky-high despite fewer COVID cases” |
| **D. Transition to the “new normal” & fading crisis salience (2023)** | Epidemiological normalization | End of crisis coverage | Shift from daily COVID heroism to structural reporting | Neutral → mildly positive | “For the first time, no COVID patients in ICU” |
|  |  | System aftershocks | Focus on waiting lists, care backlogs, fatigue | Negative | “Healthcare still suffers from pandemic aftershocks” |
|  | Social aftershocks & protest climate | Broader social discontent | Health-sector unrest embedded in wider social protest | Neutral | “Healthcare workers join nationwide protests” |

Create a codebook for the second-order axial codes providing a few sentences per quote what they revolve around and how to interpret them in a table

| **Theme** | **Axial Code** | **Analytic Description (what it revolves around)** | **Interpretive Note (how to read it)** |
| --- | --- | --- | --- |
| **A. From “heroes” to honored professionals (2020)** | **Public gratitude & symbolic appreciation** | Revolves around media and community expressions of collective admiration for nurses at the start of the pandemic — applause events, “zorghelden” labels, and public praise. It frames nursing as a moral and patriotic duty. | Indicates a strong positive sentiment peak. Symbolic recognition substitutes for structural support — emotional elevation masks underlying inequality. |
|  | **Moral elevation of bedside work** | Centers on moral framing of nurses as self-sacrificing, compassionate, and essential to national recovery. Nurses are portrayed as embodying civic virtue and care ethics. | Reflects early “hero” discourse common in crisis journalism. Useful for tracing how the press mobilizes moral archetypes to unify readers. |
| **B. Strain, burnout & workforce erosion (2020–2022)** | **Overload & emotional toll** | Revolves around depictions of exhaustion, trauma, and prolonged pressure in hospitals and care homes. The emotional labor of nurses becomes visible. | Marks a shift from admiration to empathy and concern. Media tone becomes more tragic than triumphant; emotional weight replaces heroism. |
|  | **Exodus & retention crisis** | Focuses on reports of nurses leaving the profession, unfilled training positions, and the erosion of workforce morale. | Represents structural consequences of sustained overload — not only emotional suffering but institutional fragility. Indicates transition from temporary crisis to chronic decline. |
|  | **Labor contention & collective action** | Revolves around organized protests, strikes, and collective demands for better pay and working conditions. Nurses become political actors, not only moral figures. | Frames sentiment as conflicted: public sympathy remains high, but frustration grows toward the government and institutions. Highlights the politicization of nursing identity. |
| **C. Recognition politics & contested compensation (2022–2023)** | **Policy critique & governance gaps** | Focuses on investigative and evaluative reporting on government failures: poor preparedness, late reactions, neglect of care homes. | Indicates erosion of trust in leadership. Sympathy for nurses contrasts with systemic blame — signaling a shift from individual heroism to institutional critique. |
|  | **Long-COVID recognition battles** | Revolves around compensation schemes for infected care workers; articles highlight bureaucratic injustice and union pressure for inclusion. | Expresses moral outrage on behalf of nurses. Interpreted as a justice-oriented discourse: the media reframes nurses as victims of policy failure rather than passive heroes. |
|  | **Symbolic but uneven appreciation** | Describes small gestures of gratitude (free events, bonuses) contrasted with continuing structural neglect. | Represents an ambivalent tone — recognition persists but feels hollow. Useful for identifying “performative appreciation” as a fading discourse. |
| **D. Transition to the “new normal” & fading crisis salience (2023)** | **Epidemiological normalization** | Revolves around articles announcing the end of COVID hospitalization and the return to regular care patterns. Nurses’ role recedes from front-page heroics to background stability. | Signals discursive closure of the pandemic. Positive sentiment cools to neutral — normalization replaces crisis-driven praise. |
|  | **Social aftershocks & protest climate** | Focuses on how nurses’ grievances merge with wider social unrest (inflation, protests, strikes across sectors). | Interpreted as integration of nursing discourse into broader socio-economic struggle. Shows how post-pandemic fatigue and frustration transcend the healthcare domain. |

### How the AI codes:

Initial prompt:

*You are a researcher. I have uploaded a PDF with newspaper data. You will perform what is known as qualitative coding. I have uploaded a Codebook. Use these codes. To test for intercoder reliability, I will put your inputs in ATLAS.ti. Give me the name of the article, the first word of the sentence you would code, the last word of the sentence, and what code from the codebook should be applied. Do this for the whole 86 pages. This may result in a lot of code, but that doesn’t matter. Code full sentences, so if you want to apply the code “problem solving,” give me the whole sentence, not just a few words.*

Optimize this prompt:

**Optimized Prompt**

You are a **qualitative researcher** conducting a **systematic coding analysis** of a newspaper dataset (uploaded as a PDF).
A **Codebook** has also been provided — you must use **only the codes defined in that Codebook** when analyzing the data.

Your task is to perform **qualitative coding** at the **sentence level**, following these instructions:

1. **Unit of Analysis:** Each *sentence* in the text.
2. **Coding Approach:** Apply one or more relevant **codes from the provided Codebook** to each sentence that reflects its meaning.
3. **Output Format:** For every coded sentence, include:
   - **Article Name** (as listed in the PDF)
   - **First Word** of the sentence
   - **Last Word** of the sentence
   - **Full Sentence Text**
   - **Assigned Code(s)** (exactly as labeled in the Codebook)
4. **Important Notes:**
   - Always code **entire sentences**, not phrases or fragments.
   - Apply **multiple codes** if a sentence fits more than one category from the Codebook.
   - If a sentence contains **no relevant content**, you may omit it from the output.
   - Consistency and precision are essential — your coding will be used for **intercoder reliability testing in ATLAS.ti**.
   - Accuracy is more important than brevity; a large volume of coded text is expected.

**Example Output Format**

| **Article** | **First Word** | **Last Word** | **Full Sentence** | **Code(s)** |
| --- | --- | --- | --- | --- |
| "Nurses Under Pressure" | Nurses | exhausted. | Nurses across Limburg report record levels of fatigue and emotional strain. | Workload Stress, Burnout |

Would you like me to optimize this further for **automation and scalability** (e.g., include token batching, progressive output instructions, or chunk boundaries for 86 pages)? This can make the coding process more stable for long documents

## S2. Gemini 2.5 pro outputs

### Coding tree

Initial prompt

*You are a researcher. I have uploaded 860 pages of newspaper data. You will do what is called qualitative coding. Create a coding tree that consists of 1. Open codes, group them in axial codes and group those in a few themes (3rd order codes). The codes should be able to be applied to themes that are visible in the text and revolve around the research question: How has the sentiment towards nurses, as portrayed in De Limburger’s reporting, evolved throughout the COVID‑19 pandemic (2020–2023)?*

Asked Gemini 2.5pro to optimize the prompt:
*Optimize this prompt: You are a researcher. I have uploaded 860 pages of newspaper data. You will do what is called qualitative coding. Create a coding tree that consists of 1. Open codes, group them in axial codes and group those in a few themes (3rd order codes). The codes should be able to be applied to themes that are visible in the text and revolve around the research question: How has the sentiment towards nurses, as portrayed in De Limburger’s reporting, evolved throughout the COVID‑19 pandemic (2020–2023)?*

Optimized prompt:

Here's an optimized version of your prompt, structured for clarity and to guide the AI researcher more effectively:

**Role:** You are a **Qualitative Researcher** specialized in media analysis and grounded theory.

**Data:** I have provided a corpus of 860 pages of newspaper articles from **De Limburger**, covering the period **January 2020 to December 2023**.

**Research Question:** How has the **sentiment towards nurses**, as portrayed in De Limburger’s reporting, evolved throughout the COVID-19 pandemic (2020–2023)?

**Primary Task: Thematic Qualitative Coding**

Your goal is to analyze the provided text and generate a **hierarchical coding tree** to answer the research question. This structure must follow a standard grounded theory approach:

1. **Level 1: Open Codes (In-Vivo / Descriptive)**
   - Read through the data line-by-line.
   - Identify and list specific words, phrases, or concepts (open codes) that directly describe the sentiment towards nurses or the context of their portrayal.
   - *Example codes might include: "heroes," "under pressure," "deserve a bonus," "lack of protection," "applause," "exhausted," "strike," "victim of aggression," etc.*
2. **Level 2: Axial Codes (Conceptual Categories)**
   - Group the related open codes from Level 1 into higher-level conceptual categories (axial codes).
   - These categories should define the *properties* and *dimensions* of the sentiment.
   - *Example categories might be: "Admiration & Heroism," "Working Conditions," "Public Support," "Criticism & Frustration," "Personal Toll."*
3. **Level 3: Thematic Codes (Core Themes)**
   - Synthesize the axial codes from Level 2 into a few (e.g., 3-5) overarching core themes.
   - These final themes must directly address the "evolution of sentiment" aspect of the research question.
   - *Example themes might be: "Initial Wave: Unanimous Hero-Worship," "Mid-Pandemic: Growing Strain & Public Ambivalence," "Late-Pandemic: Normalization & Systemic Critique."*

**Deliverable:**

Produce a clearly structured **coding tree** or **coding frame**. Please format it hierarchically so the relationship between Open Codes, Axial Codes, and Thematic Codes is easy to understand. The final output should be a practical tool I can use to systematically code the entire dataset.

Look back at the data see if you need to refine your codes and create a table for the codes

...

Translate the table to English

| **Core Theme (Thematic Code)** | **Axial Code (Conceptual Category)** | **Open Codes (Coded examples from the data)** |
| --- | --- | --- |
| **Theme 1: Unanimous Hero-Worship and Public Solidarity (ca. 2020)**    *(The initial, overwhelmingly positive societal reaction of admiration and gratitude.)* | **1.1: Admiration and Heroism**    *(The framing of staff as "heroes" and objects of pride.)* | "healthcare heroes", "applause", "appreciation", "proud", "gratitude", "selfless", "calling", "frontline soldiers" |
|  | **1.2: Public Support Actions**    *(Concrete, positive actions from the community to show support.)* | "surprise with pastries", "free newspapers", "card campaigns", "banners", "help virus", "offering childcare", "flowers" |
|  | **1.3: Early Recognition of Sacrifice**    *(Reporting on the personal sacrifices and dangers on the front line.)* | "fear of infection", "danger to own health", "psychological assistance", "running on fumes" (lit: "walking on their eyebrows"), "'Nobody can be a hero 24/7'", "sprinting and running a marathon" |
| **Theme 2: From Applause to Activism (ca. 2020-2021)**    *(The turning point in sentiment. The "hero" rhetoric disappears, replaced by the reality of exhaustion, system failure, and conflicts over appreciation.)* | **2.1: Physical and Mental Exhaustion (The Toll)**    *(Focus on the long-term negative impact on staff well-being.)* | "tired", "exhausted", "on their last legs", "burn-out", "mental overload", "staff can't take it anymore", "tears", "trauma", "heavy/tough" |
|  | **2.2: System Failure and Structural Shortages**    *(Reports shifting focus to the failure of the healthcare system to support its staff.)* | "staff shortage", "absenteeism", "bed shortage", "army has to help", "can't complete the roster", "waiting lists" |
|  | **2.3: Conflicts over Appreciation (Financial & Political)**    *(The struggle for tangible (financial) recognition and the perceived political unwillingness.)* | "healthcare bonus" (discussion/delay), "strike", "collective agreement negotiations", "wage increase", "political cowardice", "A bonus? Not enough" |
|  | **2.4: Public Ambivalence and Aggression**    *(The shift from applause to misunderstanding, aggression, and distrust.)* | "aggression", "short fuses", "threatened", "conspiracy theorists", "distrust", "'Corona a fairytale?'", "Where is the applause?" |
| **Theme 3: Structural Crisis and Professional Reorientation (ca. 2022-2023)**    *(The "new normal" in which the pandemic transitions into a structural crisis, and staff leave the sector.)* | **3.1: Staff Exodus and Reorientation**    *(The actual departure of staff and the motives behind it.)* | "hundreds of ICU employees quit", "Dissatisfaction drives healthcare staff... away", "Healthcare staff leave due to dissatisfaction", "nurses are leaving", "former healthcare worker starts own restaurant", "considering leaving healthcare" |
|  | **3.2: Chronic System Crisis**    *(The permanent state of crisis (shortages, waiting lists) that remained after the pandemic.)* | "'Waiting lists in healthcare are becoming chronic'", "'Crisis in healthcare is now permanent'", "'Healthcare is heading for an infarction'", "disaster train... bearing down on healthcare", "healthcare infarction" |
|  | **3.3: Struggle for Recognition (Long Covid)**    *(A new struggle, focused on recognition for occupational illness.)* | "long covid", "occupational disease", "no compensation", "Unions demand money", "GP... says goodbye: 'Concentration is gone because of corona'" |

### How the AI codes:

Initial prompt: Create a codebook for the second-order axial codes providing a few sentences per quote

what they revolve around and how to interpret them in a table

| **Axial Code** | **Description and Interpretation (How to code)** |
| --- | --- |
| **1.1: Admiration and Heroism** | This code captures the portrayal of healthcare staff as exceptional, selfless figures. [cite_start]Code any language that frames their work as a sacrifice or a calling, using terms like "helden" (heroes), "frontsoldaten" (frontline soldiers), and "onbaatzuchtig" (selfless)[cite: 168, 319, 89, 992]. Interpret this as the initial, idealized, and grateful sentiment from the public and media. |
| **1.2: Public Support Actions** | This category groups all mentions of tangible, positive actions from the community directed at healthcare staff. [cite_start]This includes organized and spontaneous acts like applause, banners, bringing pastries or flowers, offering childcare, or starting aid initiatives[cite: 125, 386, 1484, 1568]. Interpret this as the *actionable* dimension of public solidarity and gratitude. |
| **1.3: Early Recognition of Sacrifice** | This code tracks the emerging awareness that the "hero" status comes at a high personal price. [cite_start]Code mentions of the intense pressure, long hours, fear of infection, and the early need for psychological support[cite: 77, 46, 453, 618]. This often appears alongside hero-worship and foreshadows future burnout. |
| **2.1: Physical and Mental Exhaustion (The Toll)** | This code documents the severe, long-term human cost of the pandemic on the workforce. [cite_start]Code all references to staff being "moe" (tired), "uitgeput" (exhausted), "op hun tandvlees" (on their last legs), or suffering from burn-out, trauma, and psychological distress[cite: 716, 744, 1021, 1494]. This signifies the shift from "pressure" to "damage." |
| **2.2: System Failure and Structural Shortages** | This code captures reporting that shifts focus from the *virus* to the *system*. [cite_start]Code all mentions of pre-existing or acute structural problems, such as "personeelstekort" (staff shortage), "beddentekort" (bed shortage), high "ziekteverzuim" (absenteeism), and the inability to fill rosters[cite: 850, 920, 1083, 1108, 1287]. |
| **2.3: Conflicts over Appreciation (Financial & Political)** | This category documents the conflict surrounding tangible (financial and political) recognition. [cite_start]Code all discussions of the "zorgbonus" (healthcare bonus), salary demands, "cao-onderhandelingen" (collective agreement negotiations), strikes, and criticisms of "politieke lafheid" (political cowardice)[cite: 763, 912, 1027, 1070, 1326, 1337]. |
| **2.4: Public Ambivalence and Aggression** | This code captures the negative turn in public sentiment. [cite_start]Code all mentions of "agressie" (aggression), "korte lontjes" (short fuses), threats, intimidation, distrust from "complotdenkers" (conspiracy theorists), and the general fading of public support ("applaus is verstomd")[cite: 795, 1055, 1149, 1378, 1433]. |
| **3.1: Staff Exodus and Reorientation** | This code identifies the ultimate consequence of the accumulated negative factors: staff leaving the profession. [cite_start]Code mentions of employees who have "gestopt" (quit), the sector "verlaten" (left), or are actively considering it due to "onvrede" (dissatisfaction)[cite: 1021, 1455, 1457, 1551, 1660]. |
| **3.2: Chronic System Crisis** | This code describes the post-pandemic landscape where the crisis is no longer acute but chronic and structural. [cite_start]Code terms that frame the situation as a permanent state of failure, such as "zorginfarct" (healthcare infarction), "chronisch" (chronic) waiting lists, and the "ramptrein" (disaster train) heading for healthcare[cite: 1287, 1631, 1650]. |
| **3.3: Struggle for Recognition (Long Covid)** | This code focuses on the specific, tragic irony of staff who contracted "long covid" (Long Covid) on the job. [cite_start]Code all mentions of their fight for recognition as an "beroepsziekte" (occupational disease) and their struggle to get financial "compensatie" (compensation)[cite: 1519, 1609, 1616]. |

## S3. Coding tree Human

| ***Third-order codes*** | ***Second-order codes*** | ***First-order codes*** |
| --- | --- | --- |
| Initial praise | Better internal conditions | The hospital provides psychological support |
|  |  | Nurses get a say via the nurse director |
|  |  | Increased social cohesion among healthcare nurses |
|  |  |  |
|  | External support | Nurses are supported by society |
|  |  | Government praises nurses |
|  |  | Healthcare workers deserve a bonus |
|  |  | Nurses deserve to be paid more |
|  |  | Healthcare workers are heroes |
|  |  |  |
| Arising problems and frictions | Disappointment in Government | Debate and disappointment about the care bonus |
|  |  | Hospitals are opposing government decisions to protect their staff |
|  |  |  |
|  | Discrepancy between society and healthcare workers | Increased aggression in society impacts nurses |
|  |  | Hostility of society towards healthcare |
|  |  | Value statements decrease over time |
|  |  |  |
|  | Nurses are (emotionally) exhausted | Healthcare workers deserve a break |
|  |  | Healthcare workers carry a high emotional burden |
|  |  |  |
|  | Issues around vaccination | Healthcare workers are the first to get vaccinated |
|  |  | Discontent about the vaccination strategy towards nurses |
|  |  | Hospitals want to vaccinate their workers going against government policy |
|  |  |  |
|  | Care takes a toll | Postponed care has to be caught up on by exhausted staff |
|  |  | Waiting lists emerge |
|  |  | Nurse exhaustion influence quality of care |
|  |  |  |
| The new crisis | The great resignation | Absenteeism and burnout has reached an all time high |
|  |  | May nurses quit their job or work less hours |
|  |  | Psychological complaints and burnout as drivers for nurse attrition and sick leave |
|  |  | There are big problems with nursing shortages |
|  |  |  |
|  | Unease | Healthcare workers go on strike despite it being hard for them |
|  |  | Collective labour agreement negotiation |
|  |  | Nurses feel undervalued |
|  |  |  |
|  | Problem solving | Task redistribution because of nurse shortages |
|  |  | New nurse education to battle nurse shortages |
|  |  | Private health clinics help out hospitals because of their nurse shortages |

## S4. Codebook

| **Second-order codes** | **Explanation** |
| --- | --- |
| Better internal conditions | Including creating better working conditions within the hospitals or nursing homes. Both from management to nurses, but also among nurses |
| External support | Revolving around nurses being valued and seen by the government and society. This involves clapping for healthcare, the outpouring of gifts, and nurses being called heroes. |
| Disappointment in Government | Revolving around the fact that Nurses are disappointed in the Government policy, the care bonus gets delayed, and they don’t get structural pay increases. Also, the hospitals oppose government decisions because they are slow, and they want to protect their nurses |
| Discrepancy between society and healthcare workers | Including: At the start, people were applauding nurses now nurses get no praise anymore. Riots have emerged in society, and people are aggressive towards nurses. Society breaks COVID rules, further increasing the burden on the hospitals. |
| Nurses are (emotionally) exhausted | Revolving around seeing many people die and the increased hours nurses have to work takes a toll, and nurses are exhausted because there is no break |
| Issues around vaccination | Including the government's vaccination strategy to vaccinate nurses first before the elderly. Hospitals are demanding that nurses be vaccinated to work, and other issues related to vaccination. |
| Care takes a toll | Revolving around the fact that much regular care is postponed because of COVID and has to be caught up on, while nurses are still exhausted from the COVID chaos |
| The great resignation | Including all the reasons nurses are working less, so burnout, absenteeism, and quitting their jobs. |
| Unease | Revolving around the salary and collective labour agreement negotiations, involving strikes and disappointment with the government about this topic. |
| Problem solving | Revolving around all the ways hospitals solve the issues regarding nursing shortages. Including the task distribution, people outside of healthcare are getting used and the new education in the MUMC+. |

## S5. Codebook revised for AI.

Context for using the codebook: How has the sentiment towards nurses, as portrayed in De Limburger’s reporting, evolved throughout the COVID‑19 pandemic (2020–2023)?

| **Second-order codes** | **Explanation** |
| --- | --- |
| Better internal conditions | This code should be used on text that represents deliberately made changes in the internal working conditions for nurses in hospitals or nursing homes. Exclude organizations where the internal conditions improve, where nurses are not working like GPs (huisartsenpraktijken), or in hospitality. Also exclude sentences like *“Verpleegkundigen en artsen spreken van een enorme opluchting.”* Because this only speaks of improvement of conditions coming from external changes not internal changes. This code should also be used on text that represents increased social cohesion among nurses, and the hospital providing psychological support for nurses. Vaccination and task redistribution should also be excluded because they are represented in other codes. Also exclude sentences that give solutions but don’t say these are actually implemented like: “*beddentekort*  *Wat betreft de spreiding van patiënten en coördinatie valt ook nog winst te behalen, denkt hoogleraar Taco van der Vaart. “Er is nog geen geautomatiseerd systeem voor. En ik bespeur wantrouwen tussen de ziekenhuizen*.” This sentence only provides a proposed solutions but it has no implication for nurses. |
| External support | This code should be used for text that represents praise or support from people who do not have a working relationship with nurses. The external support includes clapping, nurses being seen and valued by the government and society. It also includes retired nurses or hospitality workers volunteering to help in the hospitals, the outpouring of gifts, and nurses being called heroes. |
| Disappointment in Government | Revolving around the fact that Nurses are disappointed in the Government policy, the care bonus gets delayed, and they don’t get structural pay increases. Also, the hospitals oppose government decisions because they are slow, and they want to protect their nurses. |
| Discrepancy between society and healthcare workers | Including: At the start, people were applauding nurses now nurses get no praise anymore. Riots have emerged in society, and people are aggressive towards nurses. Society breaks COVID rules, further increasing the burden on the hospitals. This code should also focus on sentences that speak to nurses feeling undervalued, or changes in societal support towards nurses. |
| Nurses are (emotionally) exhausted. | Revolving around seeing many people die and the increased hours nurses have to work takes a toll, and nurses are exhausted because there is no break. This code may look a lot like the care takes a toll code, but the key differences are that this one is a broader code that focuses on nurses being exhausted, whilst the care takes a toll code focuses solely on postponed care and the waiting list. When using the care takes a toll code for a coded sentence, don’t also use this one. |
| Issues around vaccination | In the data, there are several articles mentioning vaccines. This code focuses on all the friction that arose around nurses. It includes the vaccination strategy of vaccinating nurses before the elderly and all the unease around this. The second thing this code also represents is the issues that emerged with hospitals wanting to check if their nurses/ healthcare workers are vaccinated and demanding that they be vaccinated to work. |
| Care takes a toll | As also mentioned in the nurses are (emotionally) exhausted code, this code focuses solely on the fact that regular care is postponed because of COVID and has to be caught up on, while nurses are still exhausted from the COVID chaos. When using this code, don’t also use the nurses are (emotionally) exhausted code because this one is more specialized. Also include sentences that describe the quality of regular care going down because of the nurse's exhaustion. So to further clarify, only code when it is about regular care that is postponed because the hospital couldn’t provide it anymore because of their COVID-19 patients. Sentences like “Alle ziekenhuizen samen willen de reguliere, niet-corona-zorg zo lang mogelijk overeind houden.” Also should not be coded because they don’t say anything about the toll this takes for nurses. |
| The great resignation | This is a broad code that revolves around nurses working less. This includes nurses quitting their jobs to do other work or working fewer hours. It also includes the attrition of nurses because of burnout and them getting sick. Absence because of psychological or emotional issues is also included. However, only use this code if the text specifies these reasons for nurses working less. |
| Unease | The code unease focuses on monetary issues. This includes all the collective labour agreement negotiations and their implications for nurses. Only include nurse-related negotiations. Nurses feel undervalued (financially) and underpaid. Nurses are going on strike to demand better working conditions and express their disappointment with the government. Exclude sentences that only provide information on the hospital's financials. The Unease should focus on nurses. So, exclude sentences like “Vorig jaar zijn vooral de personeelskosten en de patiëntgebonden kosten relatief snel gestegen vergeleken met de opbrengsten”, because they do not speak on nurses. |
| Problem solving | This code revolves around all the ways hospitals solve the issues regarding nursing shortages. Including the task distribution, people outside of healthcare are getting used to the new education in the MUMC+. Exclude sentences that only talk about upscaling beds, like: “We zien het alweer snel drukker worden en zijn bezig het aantal bedden uit te breiden van 14 naar 30”, because these sentences do not say anything on nurse shortages. |

## S6. GPT-5 optimized Codebook

**🧩 SYSTEM PROMPT: Qualitative Coding for Nurse Sentiment (GPT-5)**

**ROLE:**
You are a *qualitative researcher* performing **sentence-level coding** on news articles from *De Limburger* (2020–2023).
Your goal is to identify sentences that fit specific thematic codes and output them in a structured table for later use in Atlas.ti.

**🎯 RESEARCH QUESTION**

How has the sentiment towards nurses, as portrayed in *De Limburger’s* reporting, evolved throughout the COVID-19 pandemic (2020–2023)?

**⚙️ TASK INSTRUCTIONS**

1. **Unit of analysis:**
   - Code **full sentences** (end with ., !, ?, or end of quotation).
   - Headlines are coded only if they contain an evaluative or factual statement.
2. **Output:**
   - Only include sentences that mention or clearly imply **nurses**.
   - For each coded sentence, provide:

| **Sentence** | **Applied Code(s)** | **Confidence (0–1)** | **Notes** |
| --- | --- | --- | --- |

3. **Coding logic:**
   - Apply **one main code** per sentence.
   - If two codes apply equally, list both separated by a comma.
   - If no code applies → do not output that sentence.
   - Use **confidence** 0.8–1.0 for clear matches, 0.5–0.7 for ambiguous ones.
   - If uncertain, include a note like “ambiguous between X and Y.”
4. **Priority rule (specific > general):**
   - If both EXHAUSTION and CARE_TOLL could apply → choose CARE_TOLL.
   - If both INTERNAL_IMPROVEMENT and EXTERNAL_SUPPORT could apply → choose INTERNAL_IMPROVEMENT.
   - Always prefer the **more specific** or **nurse-focused** code.

**🗂️ SECOND-ORDER CODEBOOK**

Each sentence must be matched to one of the following ten codes if applicable.

**🏥 INTERNAL_IMPROVEMENT — *Better Internal Conditions***

Implemented internal actions improving nurses’ work environment, team cohesion, or mental well-being.
**Include:** psychological support, morale initiatives, improved scheduling, internal staff programs.
**Exclude:** external praise/support, proposed but unimplemented ideas, vaccination issues, or task redistribution.
**Keywords:** “psychologische steun”, “teamgevoel”, “onderlinge band”, “nieuwe rooster”, “interne maatregelen”.
**Example:** “Het ziekenhuis biedt sinds kort psychologische begeleiding voor verpleegkundigen.”

**🙌 EXTERNAL_SUPPORT — *External Praise or Help***

Appreciation or tangible help from outside the healthcare organization.
**Include:** applause, “helden”, volunteers, government recognition, public donations.
**Exclude:** internal support actions, labor disputes.
**Keywords:** “applaus”, “helden”, “vrijwilligers”, “waardering vanuit samenleving”, “overheid waardeert zorg”.
**Example:** “Burgers applaudisseren massaal voor de verpleegkundigen.”

**🏛️ GOV_DISAPPOINTMENT — *Disappointment in Government***

Criticism or frustration with government policy or inaction toward nurses.
**Include:** delayed care bonus, lack of structural pay rise, slow response.
**Exclude:** general policy discussion without nurses.
**Keywords:** “zorgbonus”, “geen structurele loonsverhoging”, “trage besluitvorming”, “kabinet laat zorg in de kou”.
**Example:** “Verpleegkundigen wachten nog steeds op de beloofde zorgbonus.”

**⚖️ SOCIETY_DISCREPANCY — *Discrepancy Between Society & Healthcare***

Shift from societal praise to hostility or neglect toward nurses.
**Include:** loss of public support, aggression, rule-breaking increasing workload.
**Exclude:** government criticism or internal morale issues.
**Keywords:** “geen applaus meer”, “agressie tegen zorgpersoneel”, “ondergewaardeerd door samenleving”, “regels genegeerd”.
**Example:** “Eerst werden ze als helden gezien, nu krijgen ze scheldpartijen.”

**😩 EXHAUSTION — *Nurses Are (Emotionally) Exhausted***

Emotional, physical, or psychological fatigue due to workload or trauma.
**Include:** burnout, overwork, long hours, trauma, mental strain.
**Exclude:** postponed care (→ CARE_TOLL).
**Keywords:** “burn-out”, “mentaal op”, “geen rust”, “overwerk”, “uitgeput”.
**Example:** “Het vele overwerk eist zijn tol bij verpleegkundigen.”

**💉 VACCINE_ISSUES — *Issues Around Vaccination***

Sentences about vaccination strategies or tensions involving nurses.
**Include:** prioritization debates, mandatory vaccination checks, vaccine hesitancy.
**Exclude:** general vaccination of public.
**Keywords:** “vaccineren”, “vaccinatieplicht”, “verplegend personeel eerst”, “twijfel over vaccin”.
**Example:** “Het ziekenhuis wil weten wie van het personeel gevaccineerd is.”

**⏳ CARE_TOLL — *Care Takes a Toll***

Postponed or degraded regular care due to pandemic pressure on nurses.
**Include:** backlog, reduced quality, regular care collapse.
**Exclude:** tiredness without patient care effects.
**Keywords:** “reguliere zorg uitgesteld”, “achterstanden”, “kwaliteit daalt”, “te weinig personeel”.
**Example:** “De gewone zorg komt nauwelijks op gang door gebrek aan personeel.”

**🚪 RESIGNATION — *The Great Resignation***

Nurses leaving, reducing hours, or taking sick leave due to stress or burnout.
**Include:** quitting, attrition, career change, sickness due to stress.
**Exclude:** general shortages with no reason given.
**Keywords:** “verlaat de zorg”, “ziekteverzuim”, “burn-out”, “minder uren”, “uitval door stress”.
**Example:** “Steeds meer verpleegkundigen verlaten de zorg door stress.”

**💰 UNEASE — *Monetary Frustration / Labor Negotiation***

Financial dissatisfaction or pay disputes involving nurses.
**Include:** CAO negotiations, strikes, undervaluation (financially).
**Exclude:** general hospital accounting or costs.
**Keywords:** “cao”, “staking”, “loonsverhoging”, “onderbetaald”, “waardering in geld”.
**Example:** “Nurses eisen loonsverhoging in nieuwe cao.”

**🧩 PROBLEM_SOLVING — *Organizational Solutions***

Hospitals are addressing nurse shortages via redistribution or training.
**Include:** task redistribution, new training programs, reskilling, and supportive staffing.
**Exclude:** infrastructure expansion without staff measures.
**Keywords:** “taakverdeling”, “opleiding”, “ondersteunend personeel”, “maatregelen tegen tekort”.
**Example:** “Het ziekenhuis traint ondersteunend personeel om verpleegkundigen te ontlasten.”

**🧾 OUTPUT FORMAT**

Always output only coded sentences in the following Markdown table format:

| **Sentence** | **Applied Code(s)** | **Confidence** | **Notes** |
| --- | --- | --- | --- |
| "Het ziekenhuis biedt sinds kort psychologische begeleiding voor verpleegkundigen." | INTERNAL_IMPROVEMENT | 0.97 | Confirmed internal support measure |
| "Burgers applaudisseren massaal voor de verpleegkundigen." | EXTERNAL_SUPPORT | 0.99 | Societal praise |

**🧠 CODING STRATEGY SUMMARY**

- Detect sentences about **nurses or their situation**.
- Map to the **most specific** fitting code.
- Assign a **confidence score**.
- Output **only** coded sentences in the table.
- Use the provided keywords and inclusion/exclusion logic to ensure consistent labeling across articles.

## S7. Gemini 2.5 pro optimized Codebook

| **Code Name** | **Core Definition** | **Inclusion Criteria (Apply when text discusses...)** | **Exclusion Criteria (Do NOT apply when text...)** |
| --- | --- | --- | --- |
| **Better internal conditions** | Deliberate, *implemented* actions by healthcare organizations (hospitals, nursing homes) to improve the working environment for nurses. | * Increased social cohesion among nurse teams.    * Hospital-provided psychological support for nurses.    * *Actual, implemented* changes to working conditions. | * Improvements in non-nurse settings (e.g., GP practices).    * Improvements resulting from *external* factors (e.g., lower patient numbers).    * Vaccination (use Issues around vaccination).    * Task redistribution (use Problem solving).    * *Proposed* or *hypothetical* solutions that are not implemented. |
| **External support** | Positive actions or sentiments from the public or external entities (outside the healthcare organization) directed towards nurses. | * Public praise (e.g., clapping, calling nurses "heroes").    * Societal or governmental *expressions of value*.    * Volunteer help from outside the sector (e.g., retired nurses, hospitality).    * Public donations, gifts, or food sent to hospitals. | * Support from *internal* colleagues or management (use Better internal conditions). |
| **Disappointment in Government** | Nurses' (or their organizations') frustration or negative sentiment directed at government policies, (in)actions, or decisions. | * Criticism of government policy (e.g., slowness, lack of clarity).    * Delays in bonuses (e.g., "zorgbonus").    * Lack of structural pay increases from the government.    * Hospitals opposing government decisions *on behalf of* nurses. | * General societal disappointment (use Discrepancy).    * Disappointment with internal hospital management. |
| **Discrepancy between society and healthcare workers** | A perceived gap, conflict, or negative shift in the relationship between healthcare workers and the general public. | * Negative changes in public sentiment (e.g., from applause to aggression).    * Societal non-compliance with rules (e.g., breaking COVID rules) *and the stated burden this causes*.    * Public riots or aggression *directed at* healthcare.    * Nurses feeling undervalued *by society* (not just pay). | * Positive praise from society (use External support).    * Specific disappointment *with the government* (use Disappointment in Government). |
| **Nurses are (emotionally) exhausted** | General statements about nurses' physical, mental, or emotional fatigue, burnout, or exhaustion due to their work. | * Mentions of exhaustion, burnout, or being "at their limit."    * The toll of increased hours.    * The emotional toll of seeing death and suffering.    * Needing a break from the constant pressure. | * Exhaustion *specifically* and *solely* linked to postponed regular care or waiting lists (use Care takes a toll instead).    * Nurses actually quitting or taking leave (use The great resignation). |
| **Issues around vaccination** | Conflicts, debates, or logistical problems related to the COVID-19 vaccination *of nurses*. | * Debates about vaccination strategy/priority (e.g., nurses vs. elderly).    * Hospital policies requiring or checking nurse vaccination status.    * Unease or friction *among staff* regarding vaccination. | * General vaccination logistics for the *public*.    * General societal debates about vaccination *not* specific to nurses. |
| **Care takes a toll** | The specific burden, stress, or toll on nurses caused by the postponement and subsequent backlog of *regular, non-COVID care*. | * Stress from catching up on postponed care or waiting lists.    * The declining quality of *regular* care *due to* nurse exhaustion.    * The impact of waiting lists *on nurses' wellbeing*. | * General exhaustion from COVID care (use Nurses are (emotionally) exhausted).    * Statements *only* mentioning that care is postponed, without discussing the *toll on nurses*. |
| **The great resignation** | Nurses leaving their jobs, reducing their hours, or taking extended leave (attrition) *due to work-related reasons*. | * Nurses quitting their jobs (for other work or to stop working).    * Nurses deliberately working fewer hours.    * Nurse absence/attrition *explicitly linked* to burnout, sickness, or psychological/emotional issues. | * General statements about nurse shortages (use Problem solving).    * General exhaustion *without* mention of quitting or reducing hours (use Nurses are (emotionally) exhausted). |
| **Unease** | Nurse dissatisfaction related to *financial compensation* and formal *working conditions*, often leading to collective action. | * Collective labour agreement (CAO) negotiations.    * Nurses feeling underpaid or financially undervalued.    * Strikes or protests demanding better pay or conditions. | * General hospital financial issues (that don't mention nurse pay).    * Disappointment with government *policy* (use Disappointment in Government).    * Feeling *societally* undervalued (use Discrepancy). |
| **Problem solving** | *Implemented* strategies or solutions by organizations to address nursing shortages or workflow challenges. | * Task redistribution (e.g., shifting tasks to other professionals).    * Training or using non-healthcare staff for support roles.    * New educational programs to address shortages.    * Actively recruiting nurses from other sectors/countries. | * *Proposed* or *hypothetical* solutions (see Better internal conditions).    * Simply upscaling beds *without* mentioning staffing solutions.    * Financial solutions (use Unease). |
